# *In Vitro* Endothelialization Test of Biomaterials Using Immortalized Endothelial Cells

**DOI:** 10.1371/journal.pone.0158289

**Published:** 2016-06-27

**Authors:** Ken Kono, Hitomi Hiruma, Shingo Kobayashi, Yoji Sato, Masaru Tanaka, Rumi Sawada, Shingo Niimi

**Affiliations:** 1 Division of Cell-Based Therapeutic Products, National Institute of Health Sciences, Tokyo, Japan; 2 Division of Medical Devices, National Institute of Health Sciences, Tokyo, Japan; 3 Soft Materials Chemistry, Institute for Materials Chemistry and Engineering, Kyushu University, Fukuoka, Japan; Hungarian Academy of Sciences, HUNGARY

## Abstract

Functionalizing biomaterials with peptides or polymers that enhance recruitment of endothelial cells (ECs) can reduce blood coagulation and thrombosis. To assess endothelialization of materials *in vitro*, primary ECs are generally used, although the characteristics of these cells vary among the donors and change with time in culture. Recently, primary cell lines immortalized by transduction of simian vacuolating virus 40 large T antigen or human telomerase reverse transcriptase have been developed. To determine whether immortalized ECs can substitute for primary ECs in material testing, we investigated endothelialization on biocompatible polymers using three lots of primary human umbilical vein endothelial cells (HUVEC) and immortalized microvascular ECs, TIME-GFP. Attachment to and growth on polymer surfaces were comparable between cell types, but results were more consistent with TIME-GFP. Our findings indicate that TIME-GFP is more suitable for *in vitro* endothelialization testing of biomaterials.

## Introduction

Implantation of medical devices that come into contact with circulating blood is associated with the risk of coagulation and thrombosis. Indeed, contact with the material surface elicits auto-activated factor XII in blood plasma, which then cleaves prekallikrein into kallikrein, resulting in coagulation [1, 2]. In addition, adsorption of plasma proteins to the surface induces the platelet adhesion. Subsequently, adherent platelets are activated and aggregate, resulting in thrombosis. Various proteins, including fibrinogen, vitronectin, fibronectin, immunoglobulines, von Willebrand factor, high molecular weight kininogen, prekallikrein, factor XI, and factor XII are involved in this process [1, 3, 4].

To prevent these events, polymers, such as 2-methacryloyloxyethyl phosphorylcholine polymer (PMPC), poly(ethyleneglycol) (PEG), and poly(2-hydroxyethyl methacrylate) (PHEMA), have been investigated as protein-repellent surface coating. These polymers can reduce adsorption of plasma proteins, as well as suppress the denaturation of adsorbed proteins, thereby reducing coagulation and thrombosis [5–8]. In particular, PMPC has been used as coating for artificial joints, cardiovascular stents, and ventricular assist devices [9–12]. In addition, material surfaces coated with bioactive molecules such as proteins from matrix, peptides, and growth factors that enhance the attachment of endothelial cells (ECs) (i.e., endothelialization) have also been developed [13–18]. A monolayer of ECs effectively shields the surface from blood, inhibits platelet adhesion, and thus suppresses coagulation and thrombosis [19]. On the other hand, poly(2-methoxyethyl acrylate) (PMEA), a blood-compatible polymer that does not activate leukocytes, erythrocytes, or platelets *in vitro* [20], has been used to coat catheters and oxygenators [21–24]. Furthermore, because PMEA and analogous polymers were found to promote attachment of non-blood cells, they are believed to facilitate endothelialization [25].

Primary ECs have been generally used to investigate whether coated bioactive molecules can promote endothelialization *in vitro* [16–18]. However, the characteristics of these cells vary among donors, and change with time in culture [26]. Furthermore, primary cells do not proliferate indefinitely, and may therefore be unsuitable for use in standardized endothelialization tests, even though using primary ECs can be informative of differences in endothelialization among patients. Importantly, immortalized cell lines have been established by transduction of simian vacuolating virus 40 large T antigen [27] or telomerase reverse transcriptase (TERT) [28]. These cells are easy to handle, stable, and have been used in many studies.

In the present study, we used three lots of primary human umbilical vein ECs (HUVECs) and immortalized human microvascular ECs (TIME-GFP) to investigate endothelializaion on biocompatible polymers that selectively recruit ECs but exhibit antifouling activity against blood cells. The polymers consist of PMEA and its analogs poly(2-(2-methoxyethoxy) ethoxy ethyl acrylate-*co*-butyl acrylate) (30:70 mol%, PMe3A) [29], poly(tetrahydrofuran-2-ylmethyl vinyl ether) (PTHFVE), and poly(2-ethoxyethyl vinyl ether) (PEOEVE), as well as PHEMA and PMEA/PHEMA co-polymers [30]. We note that the mechanistic basis of biocompatibility is thought to be different for PHEMA than for PMEA and PMEA analogs [31]. In addition, we used TIME-GFP to perform quality inspection assays of medical devices coated with bioactive molecules that promote endothelialization.

## Results and Discussion

To evaluate the impact of various biocompatible polymers (PMEA, PHEMA, PMEA/PHEMA co-polymers, PMe3A, PTHFVE, and PEOEVE) on endothelialization, HUVECs were seeded on polymer-coated polycarbonate discs, and cell attachment and growth were evaluated. Polymer coating was confirmed by water contact angle (S1 Fig), and endotoxin content was measured to be below 0.015 EU/mL for all tested discs. We note that the U.S. Food and Drug Administration limits endotoxin content at 0.5 EU/mL. Discs were placed in PMPC-coated 6-well plates to avoid nonspecific cell attachment to the outer surface of the discs. Since primary cells such as HUVECs exhibit variability among donors, we used three lots of HUVECs from different donors (lots A, B, and C). To measure growth rate, cells were counted on day 1 and 4 after seeding, at which point cells growing in standard dish cultures would have reached confluency and proliferation slows down.

**Fig 1:**
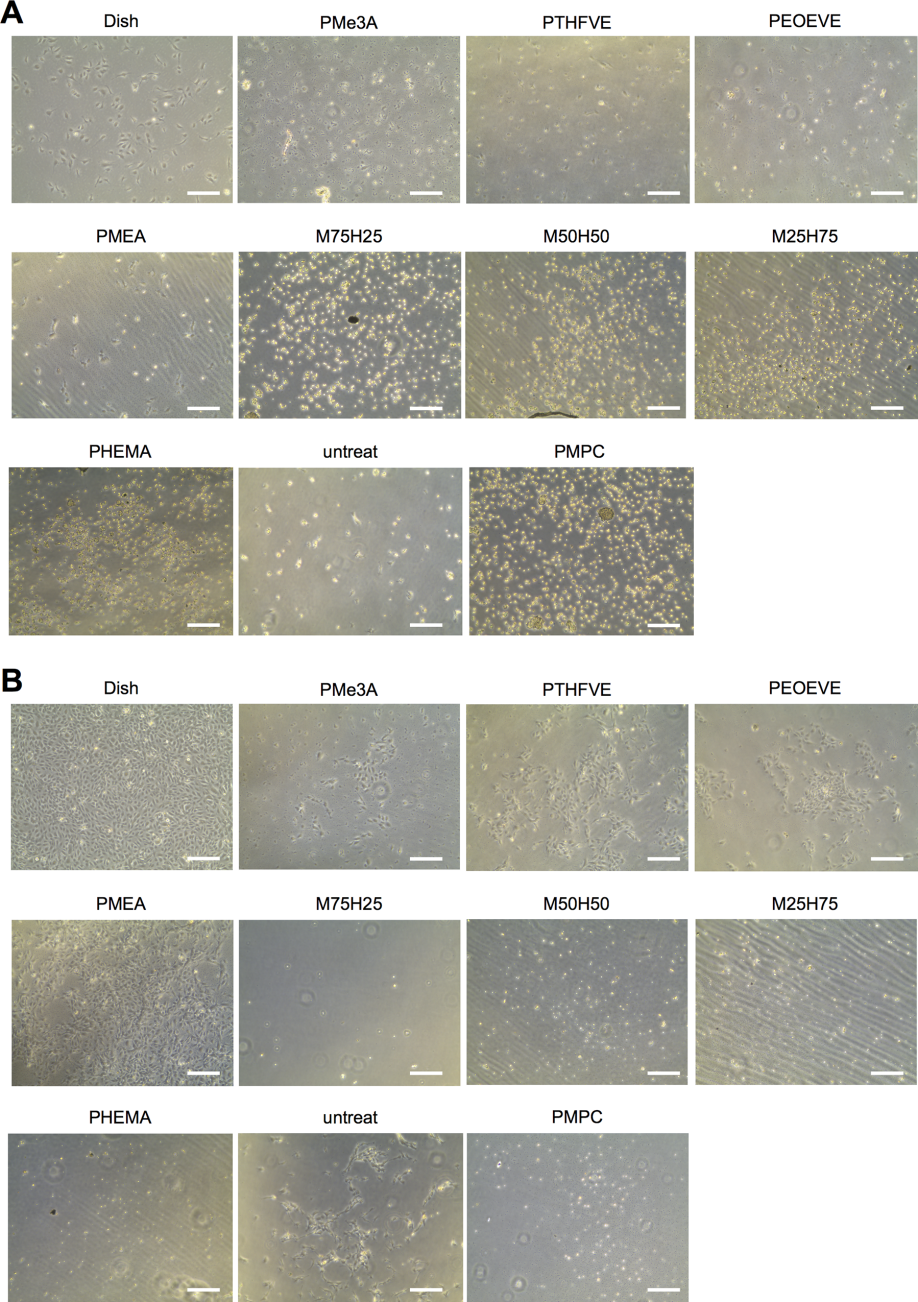
Phase contrast microscopy of HUVECs on various polymer surfaces. (A, B) Cells (6 × 10^4^) were seeded on polycarbonate (PC) discs coated with the indicated polymers (= 33 μm, thickness = 0.1 mm). The discs were placed in PMPC-coated 6-well plates. Representative images of HUVEC-A at 1 day (A) and 4 days (B) after seeding are shown. Scale bars = 300 μm. Untreat, untreated PC disc; PMPC, no PC disc.

**Fig 2:**
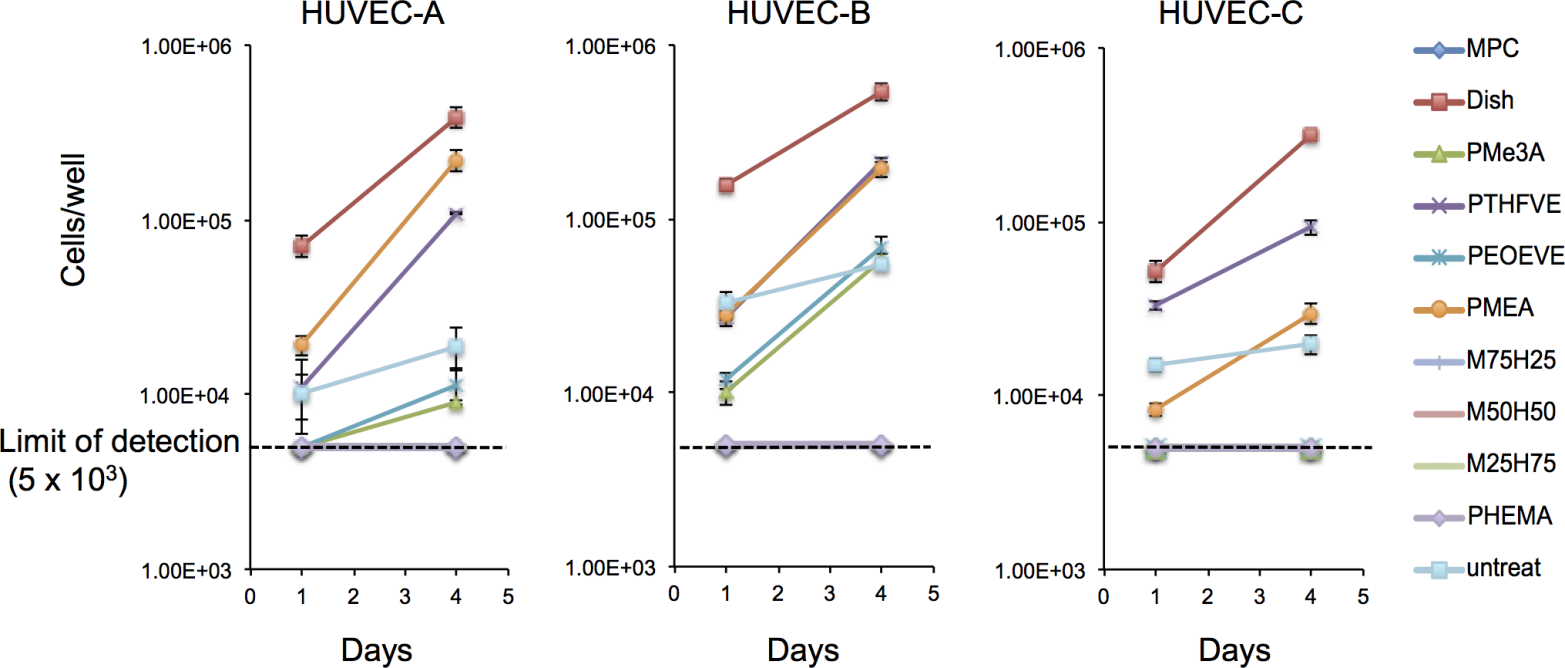
HUVEC attachment and growth profiles on various polymer surfaces. Cells (6 × 10^4^) were seeded on polymer-coated PC discs and the number of cells was counted 1 and 4 days after seeding. Results of three lots of HUVEC (A, B, and C) are presented. Error bars are standard deviations (SD) of the triplicate samples.

Representative images of lot A cells (HUVEC-A) cultured on discs for 1 and 4 days are shown in Fig 1A and 1B, respectively. Cells adhered to discs coated with PMEA but not discs coated with PHEMA or PMEA/PHEMA. Notably, adherent cells grew more vigorously than those grown in a standard culture dish (Fig 2, left panel, and Table 1). Cells growing on PMEA and PMe3A were round, whereas cells growing on PTHFVE retained normal fibroblast-like morphology similar to that of cells in dish cultures, in line with our previous reports [32, 33]. These results suggest that cells attach to PMEA and PMe3A independently of integrin, but require integrin to attach to PTHFVE. The number of attached cells was lower on PTHFVE than on PMEA (*P* = 0.01 on day 1 and *P* < 0.001 on day 4, by one-way ANOVA followed by Student-Newman-Keulis’s post-hoc test). However, cells grew equally fast on both substrates (Table 1) and there was no significant difference in viability (> 98%). Although the number of cells on PMe3A and PEOEVE was below the limit of detection (5 × 10^3^ cells/well) 1 day after seeding, a few adherent cells were observed by microscopy, and these cells grew to detectable levels 4 days after seeding. Cells generally grew faster on polymer-coated discs than on untreated discs (Table 1), and formed confluent monolayer until 7–9 days after seeding (S2 Fig), suggesting that the polymer coating promotes endothelialization. Cells grown in a PMPC-coated plate without discs did not attach, confirming that nonspecific attachment to the outer surfaces of discs was negligible.

**Table 1:** Cell growth rate on each polymer.

	HUVEC-A	HUVEC-B	HUVEC-C	Average	SD	vs. untreat, P<0.05
Dish	0.82	0.59	0.87	0.76	0.15	Yes
PTHFVE	1.11	0.99	0.50	0.86	0.32	Yes
PMEA	1.18	0.93	0.61	0.90	0.28	Yes
untreat	0.30	0.25	0.13	0.23	0.08	-
TIME-GFP	Exp1	Exp2	Exp3	Average	SD	vs. untreat, P<0.05
Dish	0.90	0.49	0.69	0.69	0.21	Yes
PTHFVE	0.87	0.71	0.53	0.70	0.17	Yes
PMEA	0.72	0.53	0.54	0.59	0.11	Yes
untreat	0.28	0.05	0.09	0.14	0.12	-

Cell growth rate (doubling/day, *R*) was calculated according to *R* = [log_2_(*N*_*4*_-*N*_*1*_)]/3, where *N*_*4*_ and *N*_*1*_ are the cell numbers on day 4 and 1, respectively. Statistical significance was determined using one-way ANOVA and Bonferroni’s post-hoc test.

The same experiment using lots B and C (HUVEC-B and HUVEC-C) did not indicate differences in cell morphology among all lots growing on any of the polymers (S3 and S4 Figs). Indeed, HUVEC-B and HUVEC-C did not adhere to PHEMA- or PMEA/PHEMA-coated discs (Fig 2), as observed for HUVEC-A. However, the number of HUVEC-B attached on PMEA and PTHFVE was comparable (*P* = 0.73 on day 1 and *P* = 0.28 on day 4), whereas the number of HUVEC-C attached was higher on PTHFVE than on PMEA (*P* < 0.001 on day 1 and *P* < 0.001 on day 4), in contrast to results for HUVEC-A. In addition, the number of HUVEC-B on PMe3A and PEOEVE was detectable 1 day after seeding, but HUVEC-C remained below the limit of detection even 4 days after seeding. These results indicate that HUVECs differ in terms of cell attachment and growth on polymers, and these differences should be taken into account when endothelialization on biocompatible polymers is evaluated using HUVECs.

**Fig 3:**
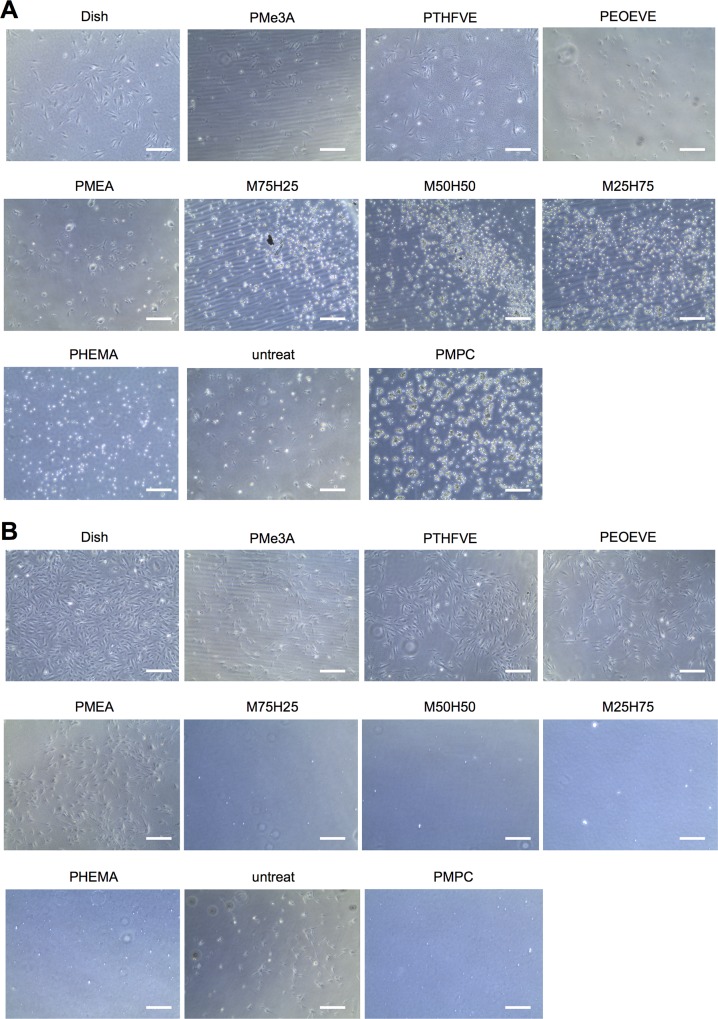
Phase contrast microscopy of TIME-GFP on various polymer surfaces. (A, B) Cells (6 × 10^4^) were seeded on PC discs coated with the indicated polymers. Representative images of TIME-GFP 1 day (A) and 4 days (B) after seeding are shown. Scale bar = 300 μm. Untreat, untreated PC discs; PMPC, no PC discs.

**Fig 4:**
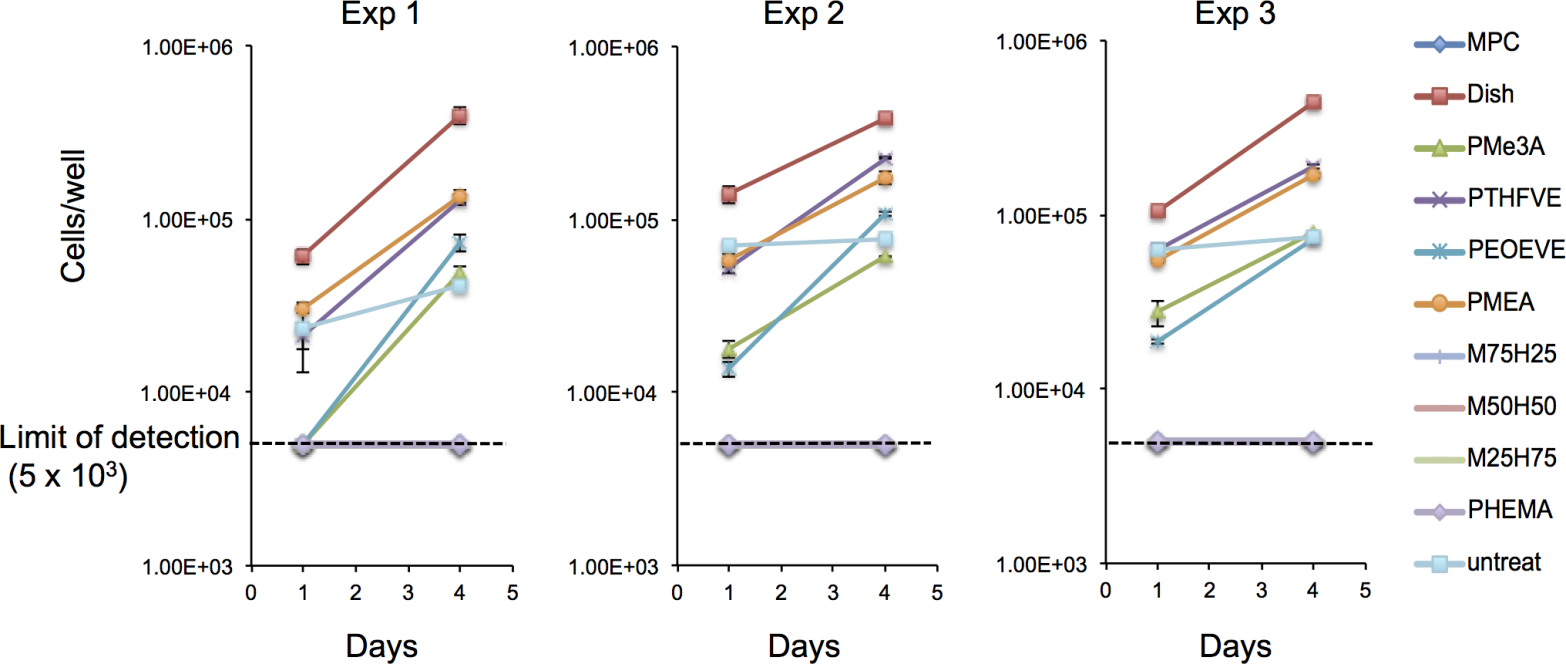
TIME-GFP attachment and growth profiles on various polymer surfaces. Cells (6 × 10^4^) were seeded on polymer-coated PC discs and the number of cells was counted 1 and 4 days after seeding. Results of three independent experiments are shown. Error bars are SD from triplicate samples.

Since HUVECs eventually stop growing with repeated passaging, the same lot of HUVECs cannot be used indefinitely. TIME-GFP is a cell line derived from human microvascular ECs immortalized with human TERT. Therefore, we investigated whether TIME-GFP can substitute for HUVECs in endothelialization tests. TIME-GFP exhibited the same morphology and attachment behavior as HUVECs (Fig 3), and the number of attached cells as well as growth rate on PMEA and PTHFVE (*P* = 0.89 on day 1 and *P* = 0.16 on day4) were comparable, as were those on PMe3A and PEOEVE (*P* = 0.76 on day 1 and *P* = 0.35 on day 4, Fig 4 and Tables 1 and 2). Notably, TIME-GFP also adhered to untreated discs, although the growth rate was slower than on polymer-coated discs (Table 1). These cells did not adhere to discs coated with PHEMA or PMEA/PHEMA, as observed for HUVECs. Taken together, the results were consistent with those obtained using HUVECs, but with less variability among experiments. We note that besides lot (donor) differences, other factors, including variability in culture media and fetal calf serum, may contribute to the variability of results using HUVECs. Nevertheless, our results indicate that using TIME-GFP and the recommended medium yields highly reproducible results.

**Table 2:** Endothelialization effects of biocompatible polymers, as measured using TIME-GFP.

			TIME-GFP	
		Attachment	Growth	NOS3, TM
PMEA	Poly(2-methoxyethyl acrylate)	++	+++	+++
PHEMA	Poly(2-hydroxyethyl methacrylate)	-	-	-
PMe3A	Poly(2-(2-methoxyethoxy) ethoxy ethyl acrylate-*co*-butyl acrylate	+	+++	+++
PTHFVE	Poly(tetrahydrofuran-2-ylmethyl vinyl ether)	++	+++	+++
PEOEVE	Poly(2-ethoxyethyl vinyl ether)	+	+++	+++

+++, comparable or superior vs dish culture; ++ and +, moderate and slight effects, respectively;–, no effect or not determined.

**Fig 5:**
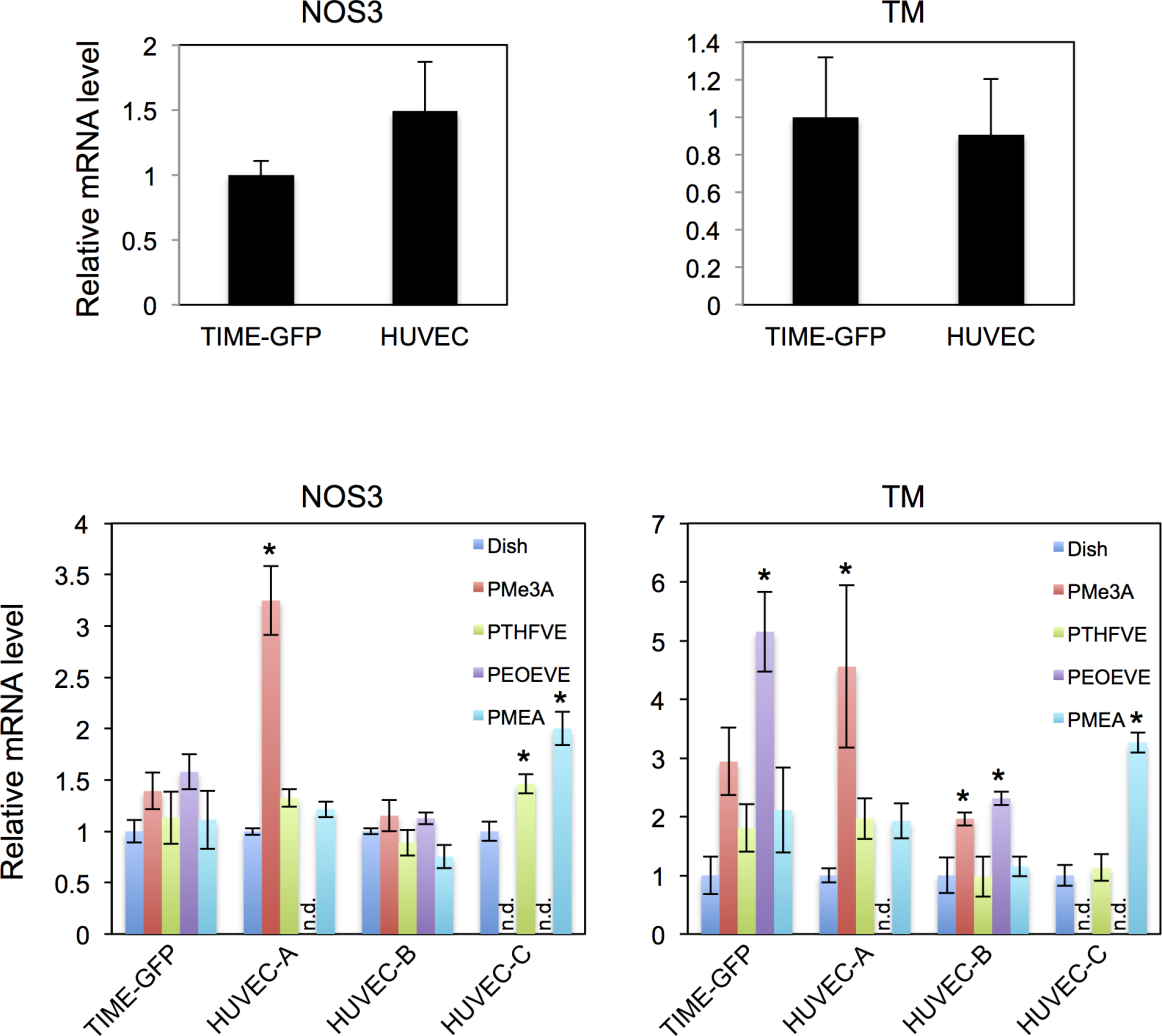
Quantitative analysis of vascular EC marker gene expression. (A) *NOS3* and *TM* expression in TIME-GFP and three lots of HUVEC grown in a culture dish for 4 days, as determined by dPCR. Results are mean ± SD of three independent experiments with TIME-GFP and three lots of HUVECs, with expression in TIME-GFP set as 1. (B) *NOS3* and *TM* expression in TIME-GFP and three lots of HUVEC grown on various polymer surfaces for 4 days, as determined by dPCR. Data are mean ± SD of three independent experiments, with expression in dish cultures set as 1. Statistical significance was determined by one-way ANOVA and Bonfferroni’s post-hoc test. *P < 0.05 vs dish culture; n.d., not determined due to insufficient total RNA.

We next examined whether TIME-GFP retains the characteristics of vascular ECs after attachment to biocompatible polymers. Thus, we measured mRNA expression of Nitric oxide synthase (NOS)3 and thrombomodulin (TM) in cells growing on polymers. NOS3 is expressed mainly in vascular ECs and synthesizes NO, which inhibits platelet aggregation [34–36]. On the other hand, TM accumulates on the surface of vascular ECs and forms a complex with thrombin to suppress blood coagulation [37, 38]. TIME-GFP and HUVECs grown in culture dishes expressed both *NOS3* and *TM* transcripts at comparable levels (*P* = 0.098 and 0.78, respectively, by unpaired Student’s t-test, Fig 5A). Furthermore, expression was not lower in cells grown on various biocompatible polymers than in cells grown in a standard dish (Fig 5B). These results indicate that *NOS3* and *TM* mRNA expression was stable in TIME-GFP grown on polymers, and that these cells retain the characteristics of vascular ECs. Moreover, the data suggest that the antithorombotic effects of endothelialization can be estimated from the number of adherent TIME-GFP.

**Fig 6:**
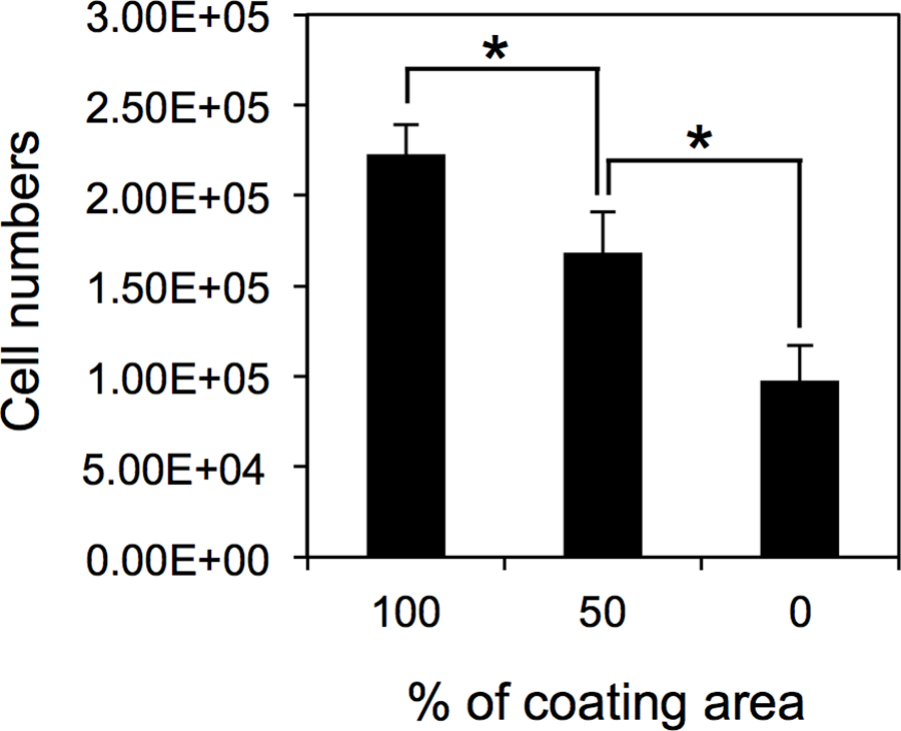
Attachment profiles of TIME-GFP on discs with 100%, 50%, and 0% PMEA coverage. *Y*-axis indicates the number of cells after 4 days of culture. Data are mean ± SD of three independent experiments. Statistical significance was determined by one-way non-repeated measures analysis of variance and the Student-Newman-Keuls’s post-hoc test (*P < 0.05).

Finally, we studied about the application of this examination. Vascular grafts that promote endothelialization have recently been developed [16, 39, 40]. In order to ensure the quality of these devices, an examination that checks the endothelialization treatment was properly done is required. Such an examination may include analysis the chemical composition of the surface. However, adherence of vascular ECs is a simpler and more direct assay. In addition, TIME-GFP may enable standardized testing without the variability inherent to primary cells. To test whether TIME-GFP can detect differences in surface treatments, we quantified the number of TIME-GFP that adhered to discs with 0%, 50%, and 100% PMEA coverage (Fig 6). Three independent experiments showed that there was a significant difference in cells attachment to discs with 100% and 50% PMEA coverage, indicating that the examination can detect defective PMEA coating, especially those coated below 50%. Thus, this assay is useful as a test of coating efficiency, although experiments with a cell line teaches us the properties of only one individual’s ECs.

## Materials and Methods

### Cells

HUVECs (lot A, 3111301; lot B, 4030901.2; lot C, 4061601.1; PromoCell, Heidelberg, Germany) were cultured in Endothelial Cell Growth Medium 2 (PromoCell). TIME-GFP, a line of immortalized microvascular ECs [American Type Culture Collection (ATCC), Manassas, VA, USA] were maintained in Vascular Cell Basal Medium (ATCC) supplemented with Microvascular Endothelial Cell Growth kit vascular endothelial cell growth factor (ATCC), 12.5 μg/ml blasticidin (Life Technologies, Carlsbad, CA, USA), and 200 μg/ml G418 (Clontech, Madison, WI, USA). Cells were maintained in a humidified atmosphere of 5% CO_2_ and 95% air at 37°C, and were passaged at 90% confluence.

### Preparation of polymer substrates

Poly(2-methoxyethyl acrylate) (PMEA), poly(2-hydroxyethyl methacrylate) (PHEMA) and methoxyethyl acrylate-hydroxyethy lmethacrylate copolymers (poly-(MEA-*co*-HEMA)) with three different compositions (75:25 mol%, M75H25, 75:25 mol%, M50H50, and 25:75 mol%, M25H75) were prepared by free-radical polymerization initiated by AIBN as described in a previous report [30]. Poly(2-(2-methoxyethoxy) ethoxy ethyl acrylate-*co*-butyl acrylate) (30:70 mol%, PMe3A) were also synthesized as described in a previous report [29]. Poly(tetrahydrofuran-2-ylmethyl vinyl ether) (PTHFVE), and poly(2-ethoxyethyl vinyl ether) (PEOEVE) were also synthesized as described in a previous report [41]. These polymers were coated on polycarbonate discs (= 34 mm, thickness = 0.1 mm, Mitsubishi Plastics, Tokyo, Japan) with spin-coating. Briefly, these polymers were dissolved in methanol at a concentration of 1% (w/v). 100 μl of each polymer solution was cast on the polycarbonate disc and spin-coated twice at 4,000 rpm for 10 sec. The polymer substrates were disinfected by exposure to UV for 15 min.

### Endothelial cell attachment and growth examination

ECs (6 × 10^4^) were seeded on polymer-coated polycarbonate discs (6 × 10^3^ cells/cm^2^), which were placed in PMPC-coated 6 well plates (Lipidure-Coat Multi-Dish A-6MD; NOF Corporation, Tokyo, Japan). Antibiotic-Antimycotic Mixed solution (Nacalai tesque, Kyoto, Japan) was added to media at seeding to a final concentration of 100 U/mL penicillin, 100 μg/mL streptomycin, and 250 ng/mL amphotericin B. After 1 and 4 days of culture, attached cells were washed with phosphate-buffered saline and harvested by treatment with 0.05% trypsin-EDTA solution (Gibco/Life Technologies, Carlsbad, CA, USA). The cells were centrifuged at 450 × *g* for 5 min and resuspended in fresh culture medium. Aliquots of suspended cells were stained with an Acridine Orange/Propidium Iodide Viability Kit (Logos Biosystems, Annandale, VA, USA) and quantified using a LUNA-FL Dual Fluorescence Cell Counter (Logos Biosystems).

### Digital (d)PCR and real time(RT-)PCR

Total RNA was extracted from 4-day cell cultures using an RNeasy Mini Kit (Qiagen, Valencia, CA, USA) following the manufacturer’s instructions, and 1 μg was used to synthesize cDNA using the ReverTra Ace qPCR RT kit (Toyobo, Osaka, Japan). The resulting cDNA was used for dPCR and RT-PCR. dPCR was performed using the QuantStudio 3D Digital PCR Master Mix (Thermo Fisher Scientific, Waltham, MA, USA) and the TaqMan Gene Expression Assay (Hs01574659_m1 for *NOS3* and Hs00264901_s1 for *TM*; Applied Biosystems, Foster City, CA, USA) on a QuantStudio 3D Digital PCR system (Thermo Fisher Scientific). *NOS3* and *TM* expression was normalized to that of *glyceraldehyde 3-phosphate dehydrogenase* (*GAPDH*). RT-PCR was performed using LightCycler Fast Start DNA Master SYBR Green I (Roche Applied Science, Indianapolis, IN, USA) and Light Cycler primer sets for *GAPDH* (Search LC GmbH Heidelberg, Germany) on a LightCycler instrument (Roche Applied Science) with associated software.

### Statistical analysis

Data were analyzed in SigmaPlot v.12.5 software (Systat Software, San Jose, CA, USA) by one-way analysis of variance followed by the Student-Newman-Keuls’s or Bonferroni’s post hoc test. *P* values < 0.05 were considered significant.

## Supporting Information

S1 Fig Static water contact angles on various polymer surfaces. Static water contact angles of examined coated-polymer surfaces tested. Data are mean ± SD of the measurements (n = 3).
(TIFF)

S2 Fig Monolayer of ECs on various polymer surfaces. HUVECs and TIME-GFP (6 × 10^4^) were seeded on PC discs coated with the indicated polymers. Discs were placed in PMPC-coated 6-well plates, and incubated until cells formed confluent monolayers. Images are representative TIME-GFP 9 days after seeding. Scale bars = 300 μm. Untreat means untreated PC disc.
(TIFF)

S3 Fig Phase contrast microscopy of HUVEC-B on various polymer surfaces. (A, B) Cells (6 × 10^4^) were seeded on PC discs coated with the indicated polymers. The discs were placed in PMPC-coated 6-well plates. Representative images of HUVEC-B at 1 day (A) and 4 days (B) after seeding are shown. Scale bars = 300 μm. Untreat means untreated PC disc.
(TIFF)

S4 Fig Phase contrast microscopy of HUVEC-C on various polymer surfaces. (A, B) Cells (6 × 10^4^) were seeded on PC discs coated with the indicated polymers. The discs were placed in PMPC-coated 6-well plates. Representative images of HUVEC-C at 1 day (A) and 4 days (B) after seeding are shown. Scale bars = 300 μm. Untreat means untreated PC disc.
(TIFF)

S1 File Supporting Materials and Methods. (DOCX)
