# Supplementary figures and images for: In Vitro Endothelialization Test of Biomaterials Using Immortalized Endothelial Cells
Source: PLoS One. 2016 Jun 27;11(6):e0158289. doi: 10.1371/journal.pone.0158289 (PMC4922589; doi:10.1371/journal.pone.0158289)

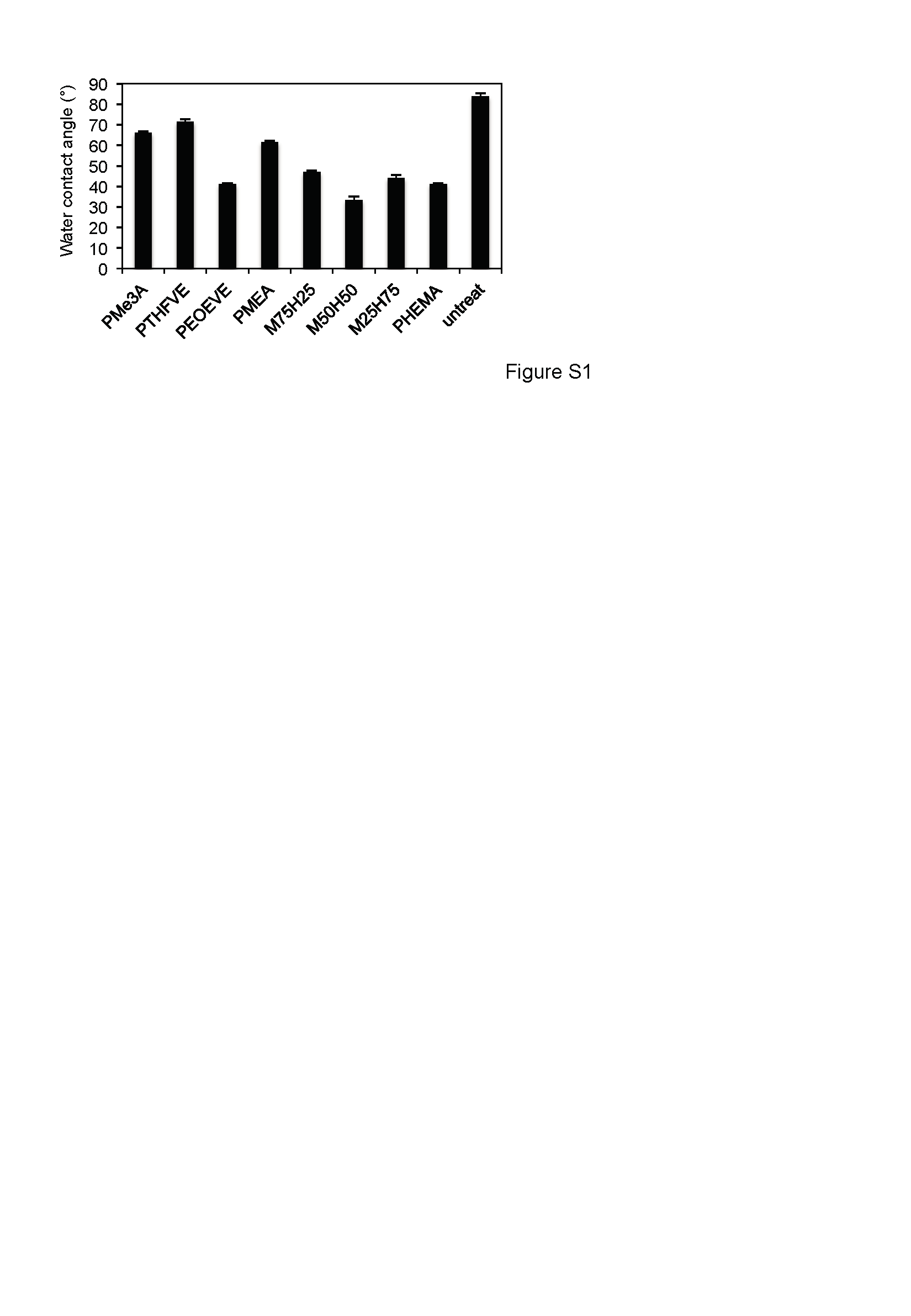

Supplement: S1 Fig — Static water contact angles of examined coated-polymer surfaces tested. Data are mean ± SD of the measurements (n = 3). (TIFF) [file pone.0158289.s001.tiff]

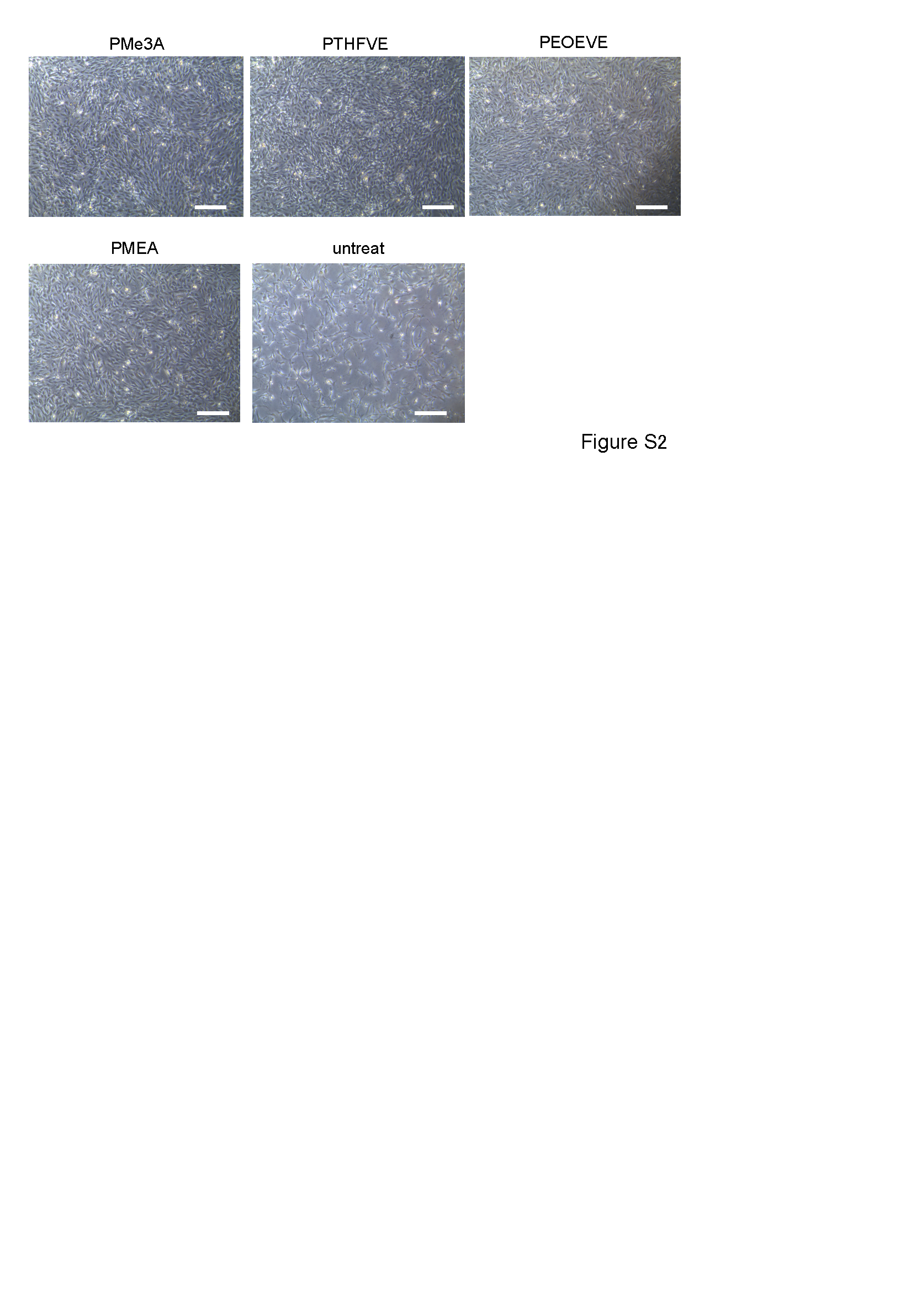

Supplement: S2 Fig — HUVECs and TIME-GFP (6 × 10^4^) were seeded on PC discs coated with the indicated polymers. Discs were placed in PMPC-coated 6-well plates, and incubated until cells formed confluent monolayers. Images are representative TIME-GFP 9 days after seeding. Scale bars = 300 μm. Untreat means untreated PC disc. (TIFF) [file pone.0158289.s002.tiff]

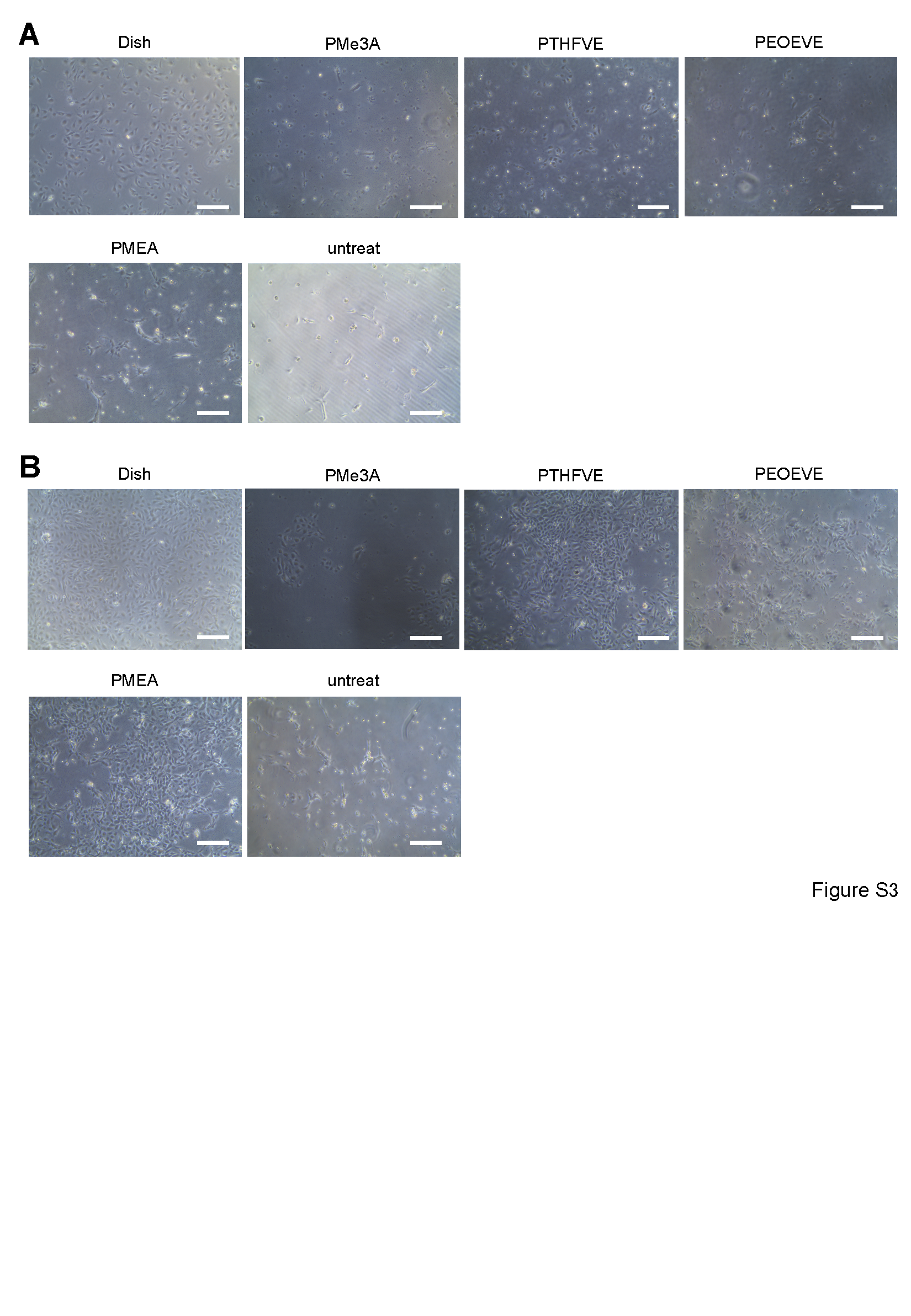

Supplement: S3 Fig — (A, B) Cells (6 × 10^4^) were seeded on PC discs coated with the indicated polymers. The discs were placed in PMPC-coated 6-well plates. Representative images of HUVEC-B at 1 day (A) and 4 days (B) after seeding are shown. Scale bars = 300 μm. Untreat means untreated PC disc. (TIFF) [file pone.0158289.s003.tiff]

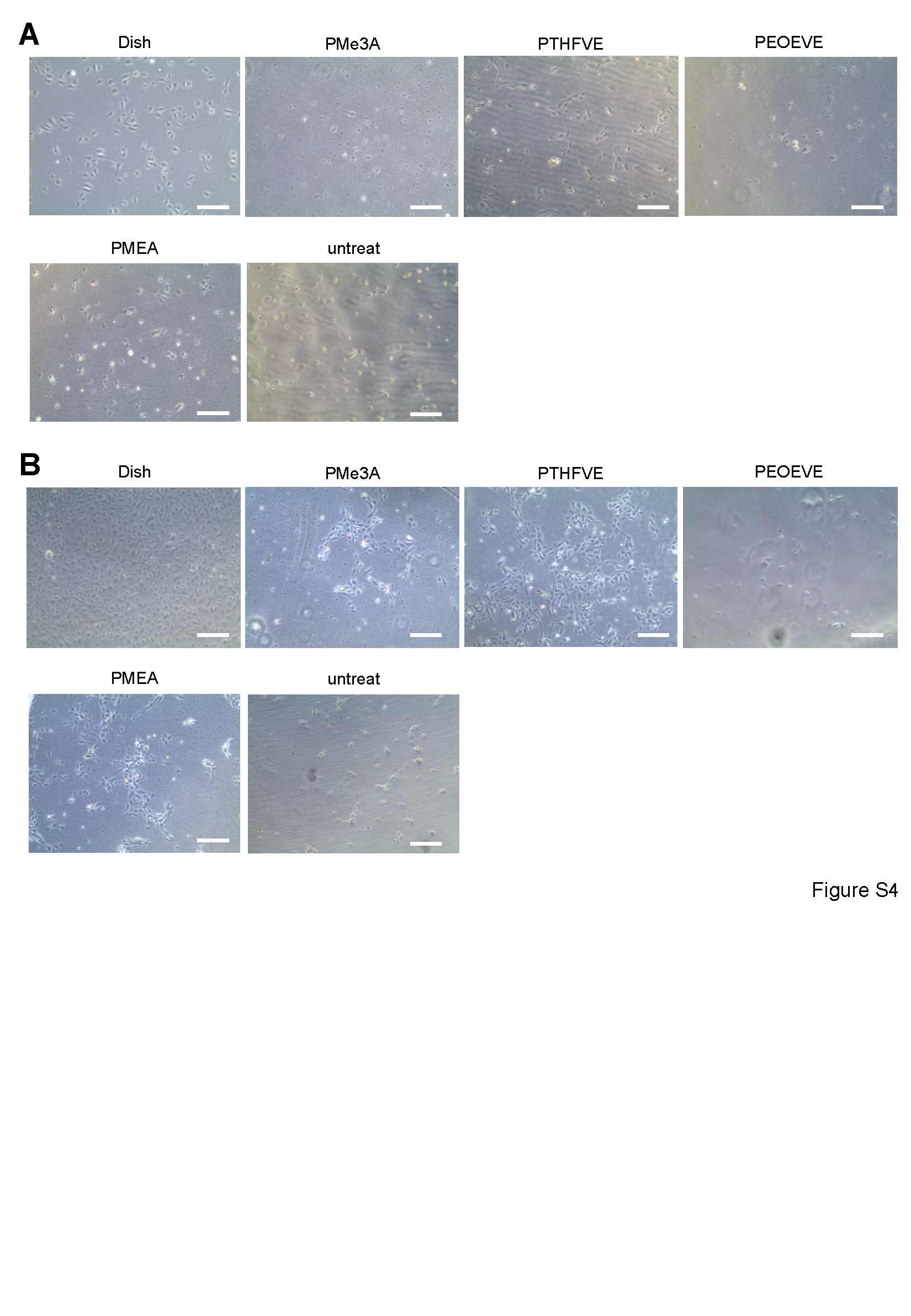

Supplement: S4 Fig — (A, B) Cells (6 × 10^4^) were seeded on PC discs coated with the indicated polymers. The discs were placed in PMPC-coated 6-well plates. Representative images of HUVEC-C at 1 day (A) and 4 days (B) after seeding are shown. Scale bars = 300 μm. Untreat means untreated PC disc. (TIFF) [file pone.0158289.s004.tiff]
