# Supplementary material for: In Vitro Endothelialization Test of Biomaterials Using Immortalized Endothelial Cells
Source: PLoS One. 2016 Jun 27;11(6):e0158289. doi: 10.1371/journal.pone.0158289 (PMC4922589; doi:10.1371/journal.pone.0158289)
Supplement: S1 File — (DOCX) [file pone.0158289.s005.docx]

**Supporting information**

**Materials and Methods**

**Static water contact angl*e***

The wettability of coated-polymer surfaces was examined by measuring static water contact angles using VCA Optima-XE (AST Products, MA, USA). Three measurements were taken on each surface at 5 sec after dropping, with Milli-Q water as probe liquid and drop size 1 µL.

**Endotoxin content**

The endotoxin content of the polymer-coated discs was determined by limulus amebocyte lysate test. The elution was prepared according to ISO10993-12, and endotoxin content was determined using Limulus ES-II Single Test (Wako, Japan) following the manufacturer’s protocol, and was confirmed to be under 0.015 EU/mL for all substrates.
